# Multi-material 3D printed composites inspired by nacre: a hard/soft mechanical interplay

**DOI:** 10.1038/s41598-025-91080-2

**Published:** 2025-02-25

**Authors:** Marco Curto, Jack Dowsett, Alexander P. Kao, Gianluca Tozzi, Asa H. Barber

**Affiliations:** 1 School of Mechanical and Design Engineering, University of Portsmouth, Portsmouth, UK; 2 School of Engineering, University of Greenwich, Chatham Maritime, UK; 3 Department of Engineering, City St. George’s, University of London, London, UK

**Keywords:** Bioinspired, Additive manufacturing, 3D printing, Composites, X-ray tomography, Multi-material, Materials science, Bioinspired materials, Mechanical engineering

## Abstract

Structural materials are used extensively in nature where mechanical function is required. These structures are composites consisting of soft and, in some cases, hard phases precisely distributed over different length scales. Bio-inspiration aims at producing materials with structure, design and/or mechanical properties adopted from biological tissues. To reproduce complex structures found in nature, additive manufacturing (AM) using three-dimensional printing (3DP) is an attractive method to assemble complex topologies with resolutions approaching the micro and nano-composition. Specifically, high-resolution MultiJetPrinting (MJP) 3D printing allows the simultaneous deposition of soft and hard photo curable plastic resins. Nacre is a prevalent example of a complex biological composite material organization that can test the ability of MJP to manufacture a bio-inspired engineering structure, where the organization of materials in nacre is optimized to avoid catastrophic failure. The ability to generate complex 3D organizations required to mimic the structure of nacre by controlled organization of soft and hard materials is achieved here using a generative design approach. Such a generative design is further enhanced by incorporating two differing MJP directions that provide relatively strong and weak interfaces between the soft and hard material phases. Consideration of classical stress transfer theory between at a hard material reinforcement interface was shown to correlate with experimental observations of mechanical performance and failure in 3D printed nacre inspired composites. Thus, the ability to distribute materials with a range of mechanical properties and incorporate further interfacial design is demonstrated. The approach presented is flexible and allows complex bio-inspired composites to be 3D printed that incorporate different interfacial quality through changing printing direction.

**Supplementary Information:**

The online version contains supplementary material available at 10.1038/s41598-025-91080-2.

## Introduction

Biological materials are widely accepted as being compositionally composites^1^, made of soft and, in some cases, hard phases assembled and precisely distributed over different length-scales^2,3^. These self-assembled composites are generated in a bottom-up fashion by nature using minimum material volumes and exploiting elements available locally^4^. Remarkable mechanical properties such as abrasion, impact, fatigue, and tolerance to crack propagation depend on the density and distribution of every element (e.g. collagen, keratin, lignin) in the composite structure.

The mechanical functions of biological composites are strictly related to stress transfer at interfaces between dissimilar phases. The properties of the interfacial are critical in defining composite properties such stiffness, viscoelasticity, strength and toughness in the same biological structure^5^. For instance, nano-dimensional fibres in cortical bone and platelets in the nacreous layer of shells provide crack control architectures with remarkable tolerance to flaws^6,7^. Fish scales, shark and human teeth are other examples of evolved composites structures organized to oppose wear and abrasion^8–11^.

The optimization of biological composites provides design inspiration for the development of engineered analogues^12^. Such design inspiration must consider the spatial distribution of materials with dissimilar mechanical properties but also the mechanical performance of resultant interfaces. The nacreous layer of shells is a significant example of strong, resilient, organic composite formed by some mollusks as an inner shell layer, protecting the soft internal organs as well as the precious and valuable pearl that is formed within oysters. The nacreous layer of shell uses a high volume fraction (95%) of ceramic (aragonite) platelets and a thin layer of protein and polysaccharide formed at interfacial regions, resulting in a non-brittle material, characterized by a work of fracture 3000 times greater than pure ceramic, and a toughness of around 3000 times higher than pure calcium carbonate^12^. Specifically, nacre microstructure consists of aragonite as hexagonal plates with extensive interfacial layers of the softer material. Nacre has intrinsic toughening mechanisms responsible of improving impact resistance over its main constituents, dissipating energy and preventing catastrophic crack propagation^13,14^. When mechanically loaded, the failure behavior of nacre relies on micro-cracking events that propagate between the mineral platelets at the interface due to a small percentage of soft bulky organic matrix that facilitates stress transfer through shear during external loading^15^. However, the precise toughening process is contentious and additional mechanisms have been proposed to be significant in contributing to its specific toughness, including crack blunting within the nacre structure, energy absorption through plate pull-out, ligament formation and extension from biopolymer failure^15,16^.

Computer aided design (CAD) and three-dimensional printing (3DP) as a physical output provide strategies to create complex topologies with features across a range of length scales^17–20^. While 3DP outputs from CAD has been extensively used to control spatial distribution of materials, the consideration of interfacial design such as the effect of varying interfacial behaviour on resultant mechanical performance is less well understood. Previous studies have used 3DP to give combinations of hard and soft materials for complex composite architectures with the potential to mimic the mechanical function found in nacre. Specifically, multi-material 3D printing technologies have been used to reproduce hybrid materials inspired by biological tissues as well as functionally graded hierarchical soft-hard composites, with ink-jetting based 3D printing notably giving nacre-inspired composites mechanics^21–28^. Composite models were previously proposed to validate the behaviour of nacre and related toughening mechanisms^24^ using Fused Deposition Modelling (FDM) printing to incorporate cohesive interfacial bonding between hard and soft phases. However, FDM technology is limited in dimensional accuracy and lacks high quality finished parts that compromises evaluation of the interfacial behaviour on resultant mechanical performance. More broadly, any composite where hard and soft materials are distributed in complex organization must incorporate interfacial design to mimic the proposes of biological composites.

The ability of 3DP to capture a variety of complex material organization to mimic designs found in nacre is enhanced by the use of Multi-Jet Printing technology (MJP) that enable manufacturing of hard and soft materials in the same assembly^25^. The ability to develop flexible digital workflows is attempted here using Generative Design (GD) to produce a range of nacre-like architectures where the importance of the interface between hard and soft materials can be evaluated. This aim is supported by mechanical evaluations and microscopy investigations to understand the interfacial failure mechanics. Specifically, mechanical testing of the resulting hybrid composites was conducted to evaluate quasi-static and dynamic performances as well as to verify pull-out of the reinforcing hard material. Subsequently, failure of the nacre-like MJP composite structures was assessed using optical imaging and, in particular, X-ray computed tomography (XCT) that provides full 3D evaluations correlating well with the 3D design processes in GD.

## Materials and methods

### Generative 3D design

**Fig. 1:**
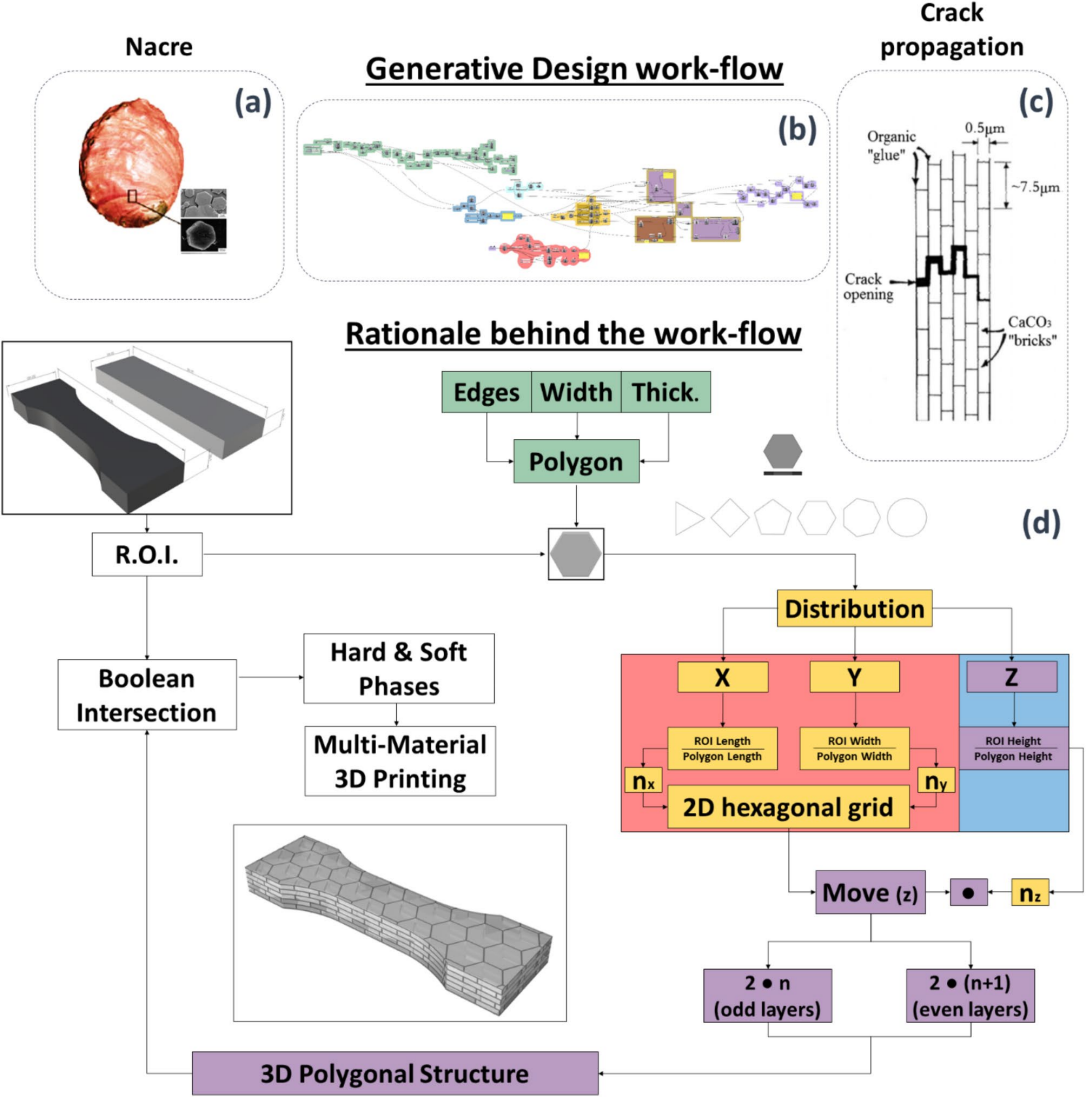
(**a**) Abalone shell with the hexagonal unit made of hard aragonite platelets within nacre (**b**) Generative design workflow (**c**) brick and mortar model with crack path deviation, (**d**) cascading automated sequence of CAD operation, implementing the generative design workflow.

A graphical algorithm editor (Grasshopper 3D, McNeel, USA), integrated with computer aided design (Rhinoceros 3D, 3D (Rhino) modelling tools (McNeel, Miami USA), was used to control the parameterization of the nacre inspired design. Principally this design considers the basic hexagonal unit of the hard aragonite platelets within nacre (Fig. 1a) and the organization of the modelled counterparts though a Generative Design workflow (Fig. 1b) into a nacre-like, brick-and-mortar model (Fig. 1c). The developed biomimetic approach integrates two different design steps: the first step produces the polygonal unit or tale in which the number of sides, diameter and thickness can be provided as input, the second step ensures the polygonal unit distribution within the boundaries of interest through a cascading automated sequence of CAD operations to generate the final design (Fig. 1d).

The hexagonal unit was repeated within the 3D space to fill the bulk of the final component by first covering a planar surface (in the X and Y axes) within the bounding box of the final part. Stacking additional layers of platelets in the Z axis up to the top surface of the bounding box (multi-material mesh). A ‘dog bone’ geometry was chosen for the bounding box. as a mechanical testing standard (ASTM D638) but with slight width modified to embed complete platelets within the volume. Length, width, and height of the bounding box were evaluated and divided by the corresponding dimensions of the designed platelet to obtain the number of grid cells n_x_ and n_y_ as shown in Fig. 1d. The grid is repeated along the Z axis as many times as allowed by the ratio between the height of the bounding box (box enclosing the object) and the height of the platelets (n_z_). Alternate layers in a ‘true-false’ pattern are shifted along the X axis so that the platelets belonging to the even layers, in the workflow of Fig. 1d indicated as 2*(*n* + 1), and the platelets belonging to the odd layers, 2*n, are always sharing the same surface area. Two offset values were introduced into the model to leave spaces in between platelets in the XY plane and Z direction. The thickness of the bulk material representing the polymer soft phase within the nacre was tuned by setting the x and y offsets representing the size of the hexagonal cell radius, and the z axis offset representing the distance between the edges of the platelets of different layers. A non-manifold merge between the bulky component and the fibres pattern creates the two regions was exported as. STL files, representing the hard phase and the complementary polymeric phase of the composite. The platelet aspect ratio was varied from 2 to 9, using a constant platelet thickness of 1 mm and platelet width ranging from 2 mm to 9 mm. The WT matrix material between the platelets was held constant at a thickness of 300 μm. These platelet dimensions resulted in a reinforcement volume fraction of the composites ranging from 53 to 65%.

### Multi-material 3D printing

An inkjet-based 3D printer (ProJet 5500X, 3D Systems, USA) was used as the manufacturing tool for the simultaneous layered deposition of a hard-white (WT) material (VisiJet^®^ CR-WT 200, 3D Systems, USA) and a soft black (BK) material (VisiJet^®^ CE-BK, 3D Systems, USA) from the same 3D printer. The 3D printer resolution was set to an ultra-high definition 13 μm layer thickness (750 × 750 × 2000 Dots per Inch (DPI)) and two orthogonal orientations of the 3D printed parts were used to ascertain variability during manufacturing. Previous work has noted the errors that can exit between the design and physically^29^.

**Fig. 2:**
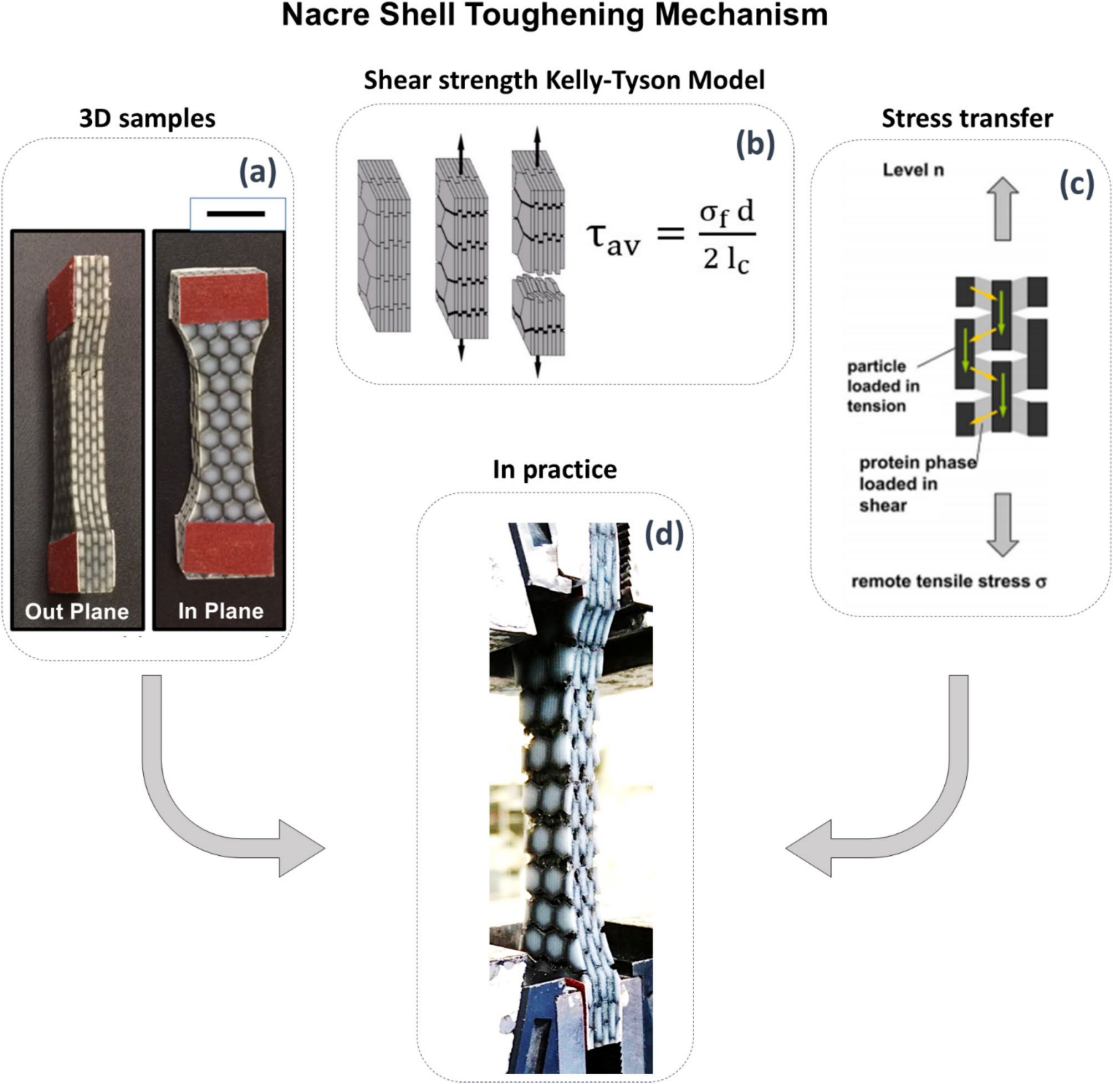
(**a**) Out of plane direction (XZ) and in-plane direction (XY), (**b**) tablet sliding mechanism at microscale justifying the relatively large strain in the case of hydrated nacre (adapted from^32^), (**c**) tension–shear model of nacre (adapted from 7), (**d**) matrix-fibres stress transfer in practice: hybrid nacre in tension while performing tensile testing.

Two sets of 3D printed hybrid nacre composites were successfully printed (Fig. 2a). The first sample set produced hard WT material platelets parallel to the 3D printing platform (in-plane), whereas the second sample set was manufactured rotated at 90 degrees to the plane of the 3D printing platform (out-of-plane). These multi-layered composites varied in terms of reinforcement volume fraction, according to the diameter of the embedded platelets. Thus, a range of nacre inspires composites, with increasing volume fraction of the reinforcement, were obtained by setting different inputs digits in the graphical algorithm editor.

The elastic modulus for WT and BK materials used for manufacturing the hybrid nacre was 364 MPa and 0.05 MPa, respectively, and were produced using the same equipment as the composite samples. The platelet aspect ratio was varied to influence the pull-out mechanism within hybrid nacre when fibre aspect ratio is varied. The stress transfer from matrix to fibre was modelled (Fig. 2b,c) using the theory of Kelly and Tyson^30^ with the assumption of a single stiff fibre comparable to the platelet reinforcement interacting with a matrix being in a plastic state. Shear transfer from the matrix to the reinforcement was observed during the tensile testing (Fig. 2d). The shear strength at the interface τ is constant and equal to the shear strength of the matrix, obtained as follow:

1 $$\:{{\uptau\:}}_{\text{a}\text{v}}=\frac{{{\upsigma\:}}_{\text{f}}\:\text{d}}{2\:{\text{l}}_{\text{c}}}$$

Where d represents the reinforcement diameter and σ_f_ is the reinforcement strength at critical length l_c_. Results from adhesive lap joint shear tests ASTM (D1002)^31^, showed an average interfacial shear strength for the BK material of 1.5 [MPa]. The platelets diameter was varied from 2 to 9 mm in steps of 1 mm, whereas the thickness of the plates was kept constant at 1 mm. The soft phase was 300 μm thick and then reduced to 30 μm, to appreciate the variation of the mechanical interplay between phases. The interaction scenario between BK and WT, evaluated through a preliminary shear test, was defined by an interfacial overlapping distance of 5 mm and a thickness of the compliant material of 300 μm. Similarly, the result of the tensile testing on WT material produced a σ_f_ value of 24.4 [MPa], whereas the considered thickness of the fibre was 1 mm.

### Optical microscopy

Optical microscopy (Leica, Switzerland) was used to evaluate the material deposition by the 3D printer and assess the quality of the manufacturing. Optical images were taken with a 25X magnification lens and the resulting platelets width and distances among platelets were measured and verified using a 0.01-millimetre division over three different locations at the ends and centre of the 3D printed samples.

### Elastic properties of composites

Elastic properties of the hybrid nacre composites were obtained following the standard ASTM D638^33^. Five samples were tested for each design condition. Mechanical tensile tests were performed at room temperature in a Universal Testing Machine (Z030, Zwick Roell, UK) fitted with a 30 kN static load cell. Samples were fully clamped and tested at a rate of 2.0 mm min^− 1^ until failure. The elastic modulus of all samples was determined according to a linear regression of the dataset according to previous work^34^. Sandpaper was applied onto the dog bone samples’ extremities to avoid sample slipping from the machine clamps.

### Impact testing

Impacting testing was used to evaluate the energy absorbing capacity of the nacre inspires composites. A particular aim here was to assess whether the composites exhibited increased energy absorption compared to the base materials, which would evidence the transfer of toughening mechanisms from nacre to the 3D printed composites. Material testing standards for additive manufacturing of polymers, ISO 179^35^, ASTM D6110^36^ and DIN EN ISO 179-1^37^ are relevant impact testing methods with the latter used in the work here. An impact tester (5102 pendulum impact tester, Zwick Roell, UK) was used in an unnotched Charpy configuration, at room temperature (21 °C). A flatwise positioning of the 3D printed parts was adopted to mimic a normal directed, physiological impact force in shell-like materials. The produced samples (80 mm×10 mm cross-section by 7 mm height) were placed in an edgewise direction and, for variety of WT volume fraction as well as in-plane and out-of-plane configurations, five samples were tested evaluating mean and standard deviation. Both nacre-like composites and base materials were tested with a strike energy of 5 J, apart from BK that was tested using a strike energy of 1 J due to its soft nature.

### In situ X-ray computed tomography (XCT)

In situ XCT was conducted using an X-ray microscope (Xradia Versa 520. Carl Zeiss Microscopy, USA) coupled with a loading device (CT5000 5KN, Deben UK Ltd). Stepwise tension was performed, and three displacement steps of 1 mm were applied; following each loading step a full tomogram was then acquired. The XCT operated at 70 kV/6 W to achieve 35 μm isotropic voxel size using a total of 1601 projections over 360°, with an exposure time of 1.5 s. The 2D X-ray projections from XCT were reconstructed to a 3D volume using the Scout and Scan Reconstructor software (Zeiss) and visualized with XRM3DViewer 1.2.8 (Zeiss).

## Results

### Elastic properties of composites

**Fig. 3:**
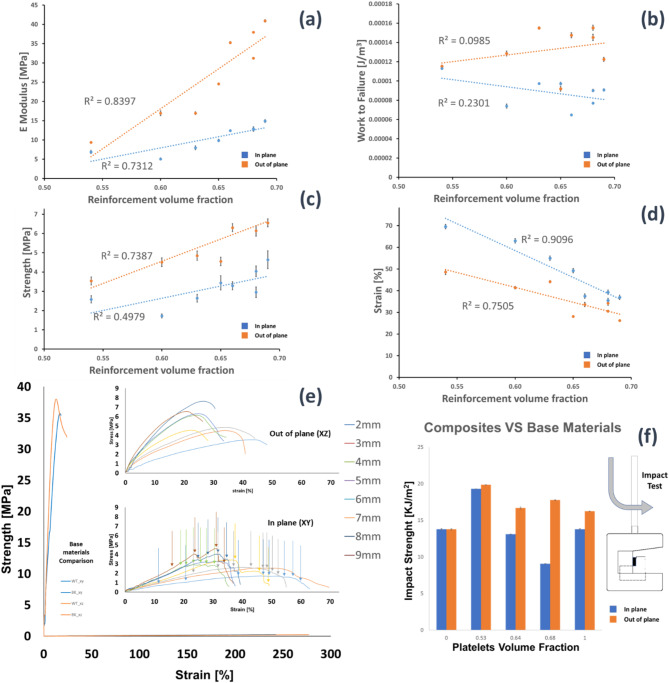
(**a**) Stiffness of in-plane and out-of-plane 3D printed nacre. (**b**) Work to fracture dependence of the reinforcement volume fraction. (**c**) Strength dependence of the reinforcement volume fraction. (**d**) Strain dependence of the reinforcement volume fraction. (**e**) Mechanical curves are similar, with in-plane 3D printed composites elongating more than the in-plane set. BK and WT in plane (xy) and out of plane (xz) curves have been reported for comparison. (**f**) Impact strength depending on platelets volume fraction.

Mechanical tensile testing of the 3D composites provided stress-strain curves as shown in Fig. 3. The initial linear response in stress strain plots gave the elastic modulus for in-plane and out-of-plane samples across the range of volume fractions examines. Out-of-plane samples showed improved elastic modulus values with increasing WT volume fraction, indicating improved reinforcement compared to in-plane samples.

**Fig. 4:**
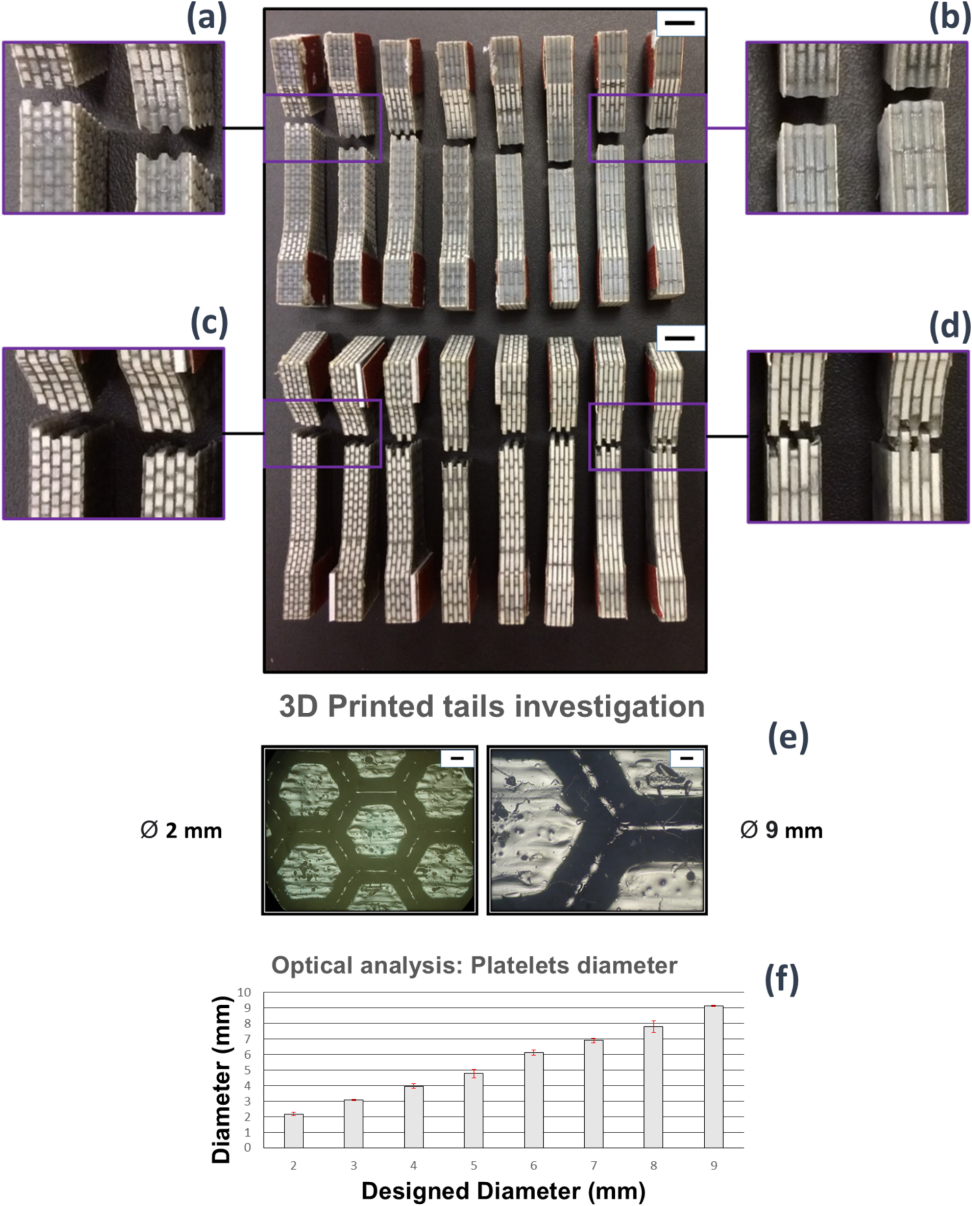
Reinforcement pull-out of both out-of-plane (top) and in-plane (bottom) 3D printed sets. (**a**) Fibre pull-out was reported by the out-of-plane set only for composites whose fibre aspect ratio was 2,3 and 4 respectively, whereas (**b**) the remaining samples broke in a brittle fashion. (**c**) Fibre pull-out was reported over the entire in-plane set and (**d**) fibres fragmentation appeared for the in-plane samples whose fibre aspect ratio was of 8 and 9, confirming the prediction of Kelly’s model, (**e**) optical images detected on 2 mm and 9 mm AM nacreous layer, (**f**) deviation of AM platelets diameters from the designed ones. The scale bar on the top right is 10 mm.

The results here demonstrate a general increase of stiffness and strength with increasing volume fraction of WT material for the out-of-plane samples as shown in Fig. 3a,c respectively compared to in-plane samples. We note that the increase in reinforcement volume fraction from 0.54 to 0.60 provides an apparently anomalous decrease in strength (Fig. 3c). The error in this measurement is relatively small, indicating that the drop in strength with this increase in volume fraction step is consistent. Such a loss in strength with increasing reinforcement volume fraction is expected to be due to a consistent manufacturing issue during the printing, probably due to the formation of the solid material at this specific volume fraction introducing defects that lower the strength of the material. Indeed, subsequent evaluations of the printed samples such as in Fig. 4 highlight imperfect interfaces that form from the manufacturing process. Toughness, such as shown in Fig. 3b for work of fracture or Fig. 3f for impact strength, shows no systematic dependence on increasing volume fraction of WT material. These results suggest that stiffness and strength behave according to typical composite behaviour where increased volume fraction of reinforcement increases stiffness and strength. The out-of-plane and in-plane composites indicate the influence of variable interfacial quality. The increase rate of stiffness and strength with WT volume fraction exhibit improved stress transfer at the interface between the WT reinforcement and BK material for the out-of-plane composites, highlighting stronger interfacial strength, compared to the less efficient interfacial stress transfer for the in-plane printed samples. The work to fracture and impact strength highlight a stronger dependence on the interfacial quality compared to the stiffness and strength results. Specifically, out-of-plane composites show increased work to fracture and impact strength due to the increase work required to fail these stronger interfaces compared to the in-plane samples. Previous work^27^ has reviewed a number of failure mechanisms in nacre, with our work suggesting that failure of stronger interfaces in out-of-plane samples causes high impact strength and work to fracture as shown in Fig. 3b,f compared to similar failure but at weaker interfaces for the in-plane samples. Other mechanisms such as increased crack deflection and stress delocalization are promoted by weaker interfaces, which are not observed here. Mineral bridges have additionally been suggested as contributing to enhanced toughening but such architectures were not incorporated into our designs.

### Impact

Results from the unnotched impact tests carried out on 3D printed out-of-plane and in-plane composites are reported in Fig. 3f. Mean values and standard deviation of the absorbed impact energy are shown for composites with increasing volume fraction of the reinforcement. The volume fraction of the reinforcement varied from 0.53 to 0.68 (20% range), with platelet width of 2 mm and 9 mm, respectively. Base materials are also reported into the bar chart with a reinforcement volume fraction of 0 for the BK bulk material (no reinforcement) and 1 for the WT brittle material (reinforcement only). In general, the two base materials gave comparable impact results. The WT (1 V_f_) material absorbed more energy than the BK (0 V_f_) when subjected to dynamic loads. Furthermore, differences between in-plane and out-of-plane printing direction are revealed only for the WT material which absorbed more energy with samples printed in out-of-plane. Conversely, the hybrid nacre composites showed good results when printed out-of-plane. In this case, all the volume fractions were able to exceed the impact strength of the base materials. This is not observed in the in-plane 3D printed hybrid nacre, which instead showed impact strength greater than base materials but only in the case of reinforcement volume fraction equal to 0.53 (platelets width 2 mm). Tensile testing was performed up to failure of the samples. Pull-out of the hexagonal platelets can be observed in Fig. 4a,b; failure through the platelet can be observed in Fig. 4c,d.

### Optical microscopy/evaluations

Optical microscopy observed holes and geometrical imperfections in both in-plane and out-of-plane cases that may contribute to premature failure when samples were subjected to tensile load (Fig. 4e,f). Surprisingly, features like edges were found between the hexagonal plates not belonging to the original CAD design. A quantitative analysis was carried out on each sample to compare platelets diameters in the 3D printed samples to the CAD design.

Figure 4f reports respective mean values and standard deviation plotted in a bar chart. Overall, platelets widths agreed with the dimension of the correspondent geometrical design testifying for the precision of the 3D printer being set at 13 μm layer thickness, producing parts with high surface quality.

### In situ X-ray computed tomography (XCT)

**Fig. 5:**
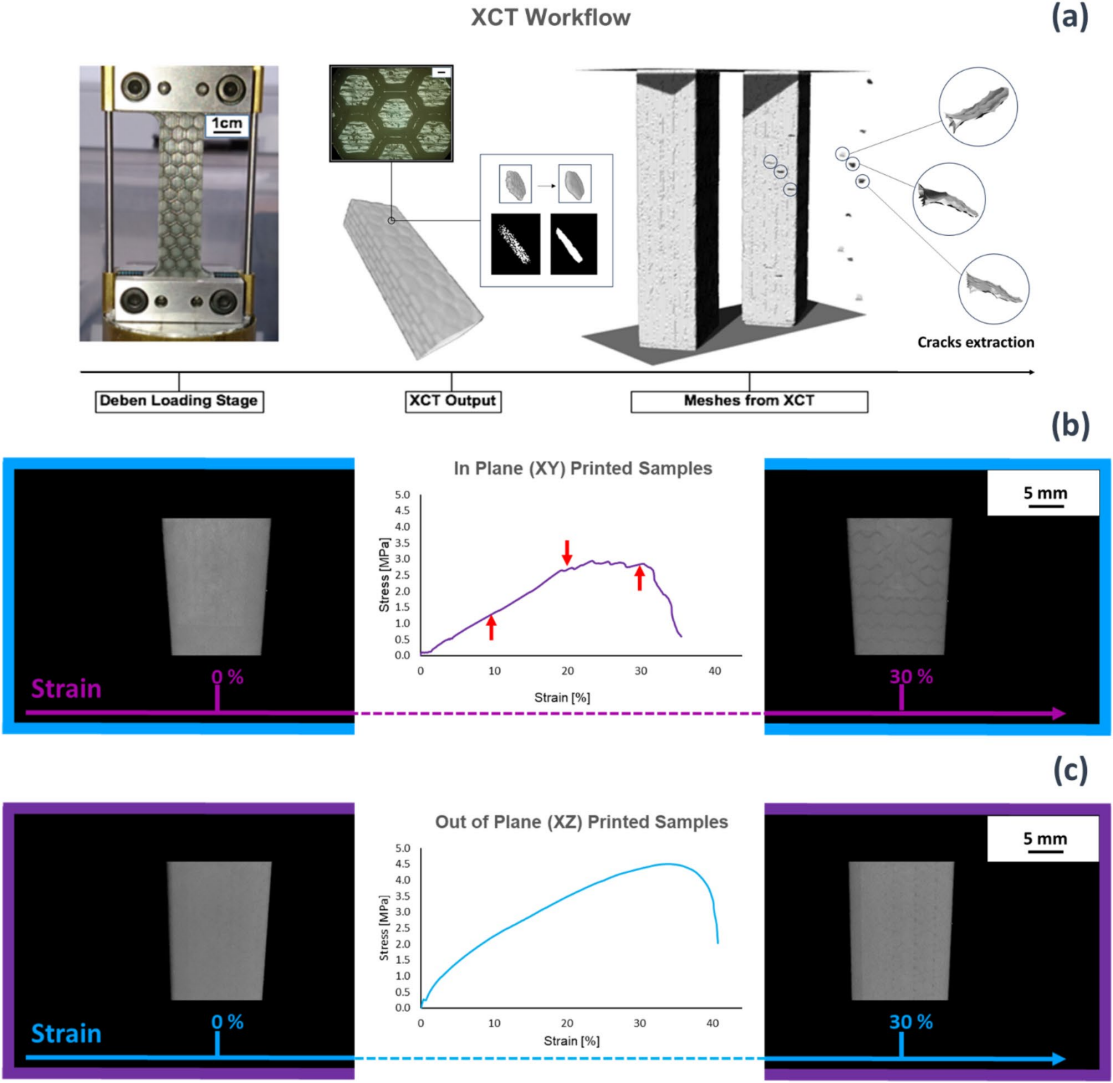
(**a**) Hybrid Nacre placed into the XCT loading stage set-up, rendering of the probed sample and platelet extraction, cracks opening when hybrid nacre is under loading conditions and cracks isolation. (**b**) XCT rendering of the in-plane 3D printed nacre accompanied by its stress-strain curve. (**c**) XCT rendering of the out of plane 3D printed nacre accompanied by its stress-strain curve.

Figure 5a shows the XCT set-up with difference between BK (mortar) and WT (bricks) assembly, where cracks and platelets are shown for representative purpose.

Despite the visibility of the two-material assembly, the reinforcement pull-out was not recorded for practical reason (hence moved and broke almost instantly) during acquisition. A visual interpretation of how cracks developed within the hybrid nacre is given in Figs. 4c and 5b where the XCT rendering of hybrid nacre in condition of zero strain is compared to 30% displacement in respect to the sample gauge length. Initiation of platelet pull-out can be clearly seen in Fig. 5b when applying low strain. The in-plane 3D printed nacre displayed a more evident crack opening compared to the corresponding sample from the out-of-plane set of samples for which cracks are less evident (Fig. 5c). Both Fig. 5b,c are accompanied by their peculiar stress strain curves. the stress strain curves recorded for in-plane and out of plane printing direction when tested in situ, which agreed with those in Fig. 5e.

## Discussion

The variation in both elastic and impact properties and, indeed, mechanical performance of a composite is defined by the interfacial behaviour between the hard and soft material constituents. Specifically, a high aspect ratio platelet will promote stress transfer from the softer matrix to the hard material; conversely, a low aspect ratio platelet will promote interfacial failure where the matrix fails in preference to platelets fracture. The elastic properties of the 3D printed composites in this work all showed increases in stiffness and strength with increasing volume fractions of platelets as expected. Improvements in the elastic properties for the out of plane set compared to the in-plane one, across the whole reinforcement volume fraction range, suggest that volume fraction alone is not sufficient to explain overall mechanical performance. Indeed, the change in printing direction clearly indicate that improved stress transfer is occurring for the out of plane printed composites compared to the in-plane composites.

**Fig. 6:**
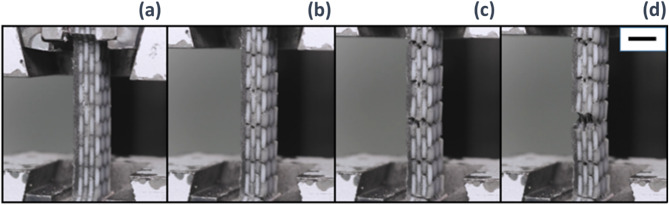
A pictures sequence showing the evolution of the cracks over the hybrid nacre structure embedded in the dog bone sample used for the tensile tests. (**a**) An external load is applied to the composite. (**b**) Cracks appear due to matrix failure. (**c**) Reinforcement pull-out initiates, demonstrating that the shear stress transmitted to the reinforcement did not exceed the reinforcement tensile strain. (**d**) Cracks grow sufficiently to promote catastrophic failure with reinforcement pull-out. The reported scale bar is 5 mm long.

Under loading, the propagation of cracks manifested within all the 3D printed samples for both in-plane and out-of-plane printing direction. The black elastomeric matrix was able to transfer enough shear stress to the reinforcement without exceeding the platelet tensile strain. In other words, the critical length was not exceeded, and the platelet reinforcement pull-out was achieved. Although the in-plane composites work to fracture absorbed less energy (Fig. 3b), observations confirmed extensive crack deflection development. Similar conclusions on reinforcement volume fraction were found in a previous study^25^ where nacre-like specimens with brick and mortar microstructures were fabricated with dual-material 3D printing technology. As per the current study, a brittle rigid material and a rubber-like material were selected; the elastic moduli of the rigid glassy polymer (VeroWhitePlus) and the rubbery polymer (TangoPlus) were 1250 MPa and 0.4 MPa, compared to 364 MPa and 0.05 MPa in this study. Despite the mismatch in terms of base materials, similar conclusions related to the brick’s aspect ratio were found. Specifically, a high aspect ratio promoting stress transfer from the matrix though the reinforcement and maximising the probability of brittle failure of the composite; a low aspect ratio promoting interfacial failure where the continuous matrix yields before the platelets fail^25^. Trialling different 3D printing directions confirmed a dependency in terms of mechanical properties from the 3D printing direction; from a dynamic standpoint, both 3D printing directions can produce composites that are tougher than the base materials (Fig. 3f). Out-of-plane hybrid nacre absorbs more energy, overcoming the impact resistance of base materials. This observation can be explained by the impact results from the WT base material which is 15% tougher when printed out-of-plane than in-plane. The consequential material mixing results in a stronger interface when hard and soft material are 3D printed, able to transmit more shear stress from matrix to reinforcement. This result confirms why out-of-plane hybrid nacre showed a brittle failure when subjected to axial loads, restricting the cracks propagation to 4 mm wide platelets (Fig. 4a,b) contrary to the in-plane set for which the critical length has been confirmed being 8 mm as shown in Fig. 6a–d.

A successful 3D design of nacre has been developed through a generative algorithm showing the ability of parametric design with CAD. The 3D modeller has allowed the user to draw shapes and geometries according to the desired design concept. Questions about the printability of generated files, mechanical interplay between hard and soft phases as well as the mechanical behaviour of the hybrid composite under static and dynamic loading conditions arise from this iterative way of designing. A similar study^38^ presented a novel design approach for a 3D printed nacre-like composite model that considered several nacreous features, such as a Voronoi shaped arrangement, tablet cohesion and interlayer adhesive. In that study, a geometric mapping approach was adopted to produce nacre mimetic composites to be fabricated with a modern biomaterial additive manufacturing technique. Despite the detailed mathematical description of the rationale behind the design generation, the outlined method seemed to be less user friendly when compared to the GD adopted in the current study. Indeed, the strength of GD and algorithm modelling represent an innovative tool that aims to speed up the design process due to its graphical or visual approach. Moreover, 3D printing technology offers prospects for cost-effective and large-scale manufacturing of nacre–like composites^38^ which is in line with the GD design principles that, being user friendly, can drastically reduce experimental testing and redesigning, fostering design fabrication and manufacturability.

In the current study, the platelet-based hybrid structure did not incorporate any feature responsible for toughening mechanisms within biological nacre such as dovetails, mineral bridges, and nano-asperities. A simple brick and mortar structure, able to define a lower bound for the entire composite interfacial hardening, was adopted and explored during quasi-static loading conditions. However, nacreous structures primary function is to resist dynamic loading conditions from impact and dropped weights. It was observed that the identified critical length applied only to the in-plane set, that showed platelet reinforcement pull-out within samples as predicted by the Kelly-Tyson model. Critically, an enhanced stress transfer from matrix to fibres was reported in the out-of-plane 3D printed set, where the fracture of the reinforcement plates occurred within samples with an *l*_*c*_ equal to 4 mm. The whole in-plane 3D printed set revealed the pull-out mechanism, whereas the out-of-plane set revealed some pull-out but only for the 2 mm to 4 mm platelets width. The ideal case would have seen platelets fracture for aspect ratio equal or greater than 8. Nevertheless, an aspect ratio pull-out threshold was presented by the out-of-plane 3D printed set for which platelet reinforcement with aspect ratio larger than 4 broke, rather than pulling-out from the matrix. This observation suggested how weaker interfaces emerged from the in-plane (XY) 3D printing that exhibited less mechanical interplay in comparison to those produced out-of-plane (YZ). Such interfacial failure could be attributed to a stronger materials bonding when composites are printed out-of-plane given the presence of both WT and BK materials in every UV cured layer. The ability of hybrid nacre is to absorb more energy than the base materials when subjected to dynamic loads. Especially for the case of out-of-plane 3D printed composites, all the volume fractions were able to exceed the impact strength of the base materials. This impact strength was not observed in the case of the in-plane 3D printed composites, where a superior impact strength was shown only in the case of composites with a platelet’s width of 2 mm. Mechanical evaluations of the 3D printed structures and their effectiveness to be tough under dynamic loading conditions warrant further studies. The XCT (Fig. 5b,c) highlighted the crack opening and propagation within in-plane and out-of-plane manufacturing. The associated stress-strain curves (Figs. 4c and 5b) reflected the shear force transfer within the sample, and are consistent with Fig. 3e, reflecting the tough pull-out mechanism distinctive of the in-plane fabrication and confirming the importance of printing direction in the mechanical properties of the builds. A critical aim of the evaluations is the potential for XCT to distinguish between different UV curable 3D printing materials but similar in density, hence in X-ray attenuation. In a recent study^29^, XCT was employed to qualitatively and quantitatively analyse the assembly of ink-jet multi-material AM composites made of soft and hard AM polymeric phases. The image processing workflow based on the region growing procedure was implemented in this study to quantitatively analyse the surface area and volume of the platelets directly assembled in the nacre-like composite by the ink-jet 3D printer. The XCT workflow in Fig. 5a, highlights the segmented hexagonal tail. The volume of the tails was almost replicated with the digital design, showing a maximum volume reduction of 0.12% in comparison to the original CAD drawing whereas the scanned surface area slightly differed from the design, showing a maximum of 7% surface area increase. Additional information about the mechanics of the hybrid nacre is needed to contextualise the use of appropriate multi-material 3D printing technology to realise nacre-like composites, and related toughening mechanisms, in one single manufacturing process. Optical observations in Fig. 4 support the importance of interfacial control in the failure of the 3D printed nacre-like composites. Composites were printed here with a range of platelet length to diameter ratios, which in a 2D cross-sectional representation gives an aspect ratio. Samples with aspect ratios of 4 and below clearly demonstrated platelet failure and pullout, such as shown in Fig. 4a. Aspect ratios of 8 and above exhibited platelet failure in Fig. 4d where the stress transfer was sufficient to fail the reinforcement. The Kelly-Tyson model is noted as a simple 2D representation of a composite cross-section in Eq. 1. Assuming the platelet geometry in this same cross-sectional representation gives a platelet width that is comparable to the length, where the critical length lc fails the reinforcing platelet, and the reinforcement diameter d. Numerically calculating the critical fibre length lc for the platelets in the 3D printed nacre-like composite using a platelet strength of 24 MPa, a platelet diameter of 1 mm as used in all designs and interfacial shear strength of the BK material, from^38^ as 1.5 MPa as stated above, gives a critical platelet width 8.1 mm or aspect ratio of 8.1. This simplification acknowledges that the hexagonal shape provides additional complexity to the stress transfer mechanisms as a 3D problem. However, the simple application of a Kelly-Tyson approach indicates sufficient stress transfer to failure the platelet reinforcement in the 3D printed composites at the corresponding aspect ratio of 8. The observations in Fig. 4d qualify the validity of the Kelly-Tyson model in predicting the condition of sufficient stress transfer to failure the platelets in the 3D printed nacre-like composites of this work.

## Conclusion

A physical 3D printed nacre design was successfully manufactured adopting a biomimicry workflow. The high controllability of parametric modelling and GD over the hybrid nacreous design enabled fabrication of objects that can be parameterised and customised in function of the final application. Kelly-Tyson composite model was used to derive the interfacial shear strength between reinforcement platelets and matrix, in relation to the mechanical properties of the employed materials. A critical reinforcement length was identified as dimensional parameter below which, using WT material for the reinforcement and BK for the matrix, interfacial failure between matrix and reinforcement was promoted to enhance nacre-like energy dissipation mechanism to avoid brittle fracture of the composite. In-plane and out-of-plane 3D printed nacre–like structures were fabricated and further tested under static and dynamic loading. The identified critical platelet reinforcement length applied only for the in-plane set, which presented pull-out within samples when the platelet reinforcement aspect ratio was lower than the threshold predicted by Kelly-Tyson. An enhanced stress transfer from matrix to platelet reinforcement was reported in the out-of-plane 3D printed set, where the fracture of the reinforcement occurred within samples. In general, nacre samples manufactured in-plane exhibited less mechanical interplay in comparison to those produced out-of-plane, for which all the volume fractions were able to exceed the impact strength of the base materials. This increased impact strength was absent in the case of the in-plane 3D printed composites.

## Electronic Supplementary Material

Below is the link to the electronic supplementary material.

Supplementary Material 1

## Data Availability

The datasets generated during the current study are available from the corresponding author on reasonable request.

## References

[1] Dunlop, J. W. C. & Fratzl, P. Biological composites. *Ann. Rev. Mater. Res.* **40**, 1–24 (2010).

[2] Fratzl, P., Dunlop, J. W. C. & Weinkamer, R. *Materials design inspired by nature: Function through inner architecture* (Royal Society of Chemistry, 2013).

[3] Wainwright, S. A. *Mechanical design in organisms* (Princeton University Press, 1982).

[4] Fratzl, P., Dunlop, J. W. C. & Weinkamer, R. *Materials design inspired by nature: Function through inner architecture*. *RSC Smart Materials* **1**, (Royal Society of Chemistry, 2013).

[5] Barthelat, F. Designing nacre-like materials for simultaneous stiffness, strength and toughness: Optimum materials, composition, microstructure and size. *J. Mech. Phys. Solids* **73**, 22–37 (2014).

[6] Ritchie, R. O. The conflicts between strength and toughness. *Nat. Mater.* **10**, 817–822 (2011). 22020005 10.1038/nmat3115

[7] Gao, H., Ji, B., Jager, I. L., Arzt, E. & Fratzl, P. Materials become insensitive to flaws at nanoscale: Lessons from nature. *Proc. Natl. Acad. Sci.* **100**, 5597–5600 (2003). 12732735 10.1073/pnas.0631609100 PMC156246

[8] Wang, L., Song, J., Ortiz, C. & Boyce, M. C. Anisotropic design of a multilayered biological exoskeleton. *J. Mater. Res.* **24**, 3477–3494 (2009).

[9] Barani, A. et al. Mechanics of longitudinal cracks in tooth enamel. *Acta Biomater.* **7**, 2285–2292 (2011). 21296195 10.1016/j.actbio.2011.01.038

[10] Enax, J., Janus, A. M., Raabe, D., Epple, M. & Fabritius, H. Acta Biomaterialia Ultrastructural organization and micromechanical properties of shark tooth enameloid q. *Acta Biomater.* **10**, 3959–3968 (2014). 24797528 10.1016/j.actbio.2014.04.028

[11] Ashby, M. F. *Materials selection in mechanical design* (Elsevier Science, 2004).

[12] Whitesides, G. M. Bioinspiration: Something for everyone. *Interface Focus* **5**, 6–7 (2015). 10.1098/rsfs.2015.0031 PMC4590425 26464790

[13] Barthelat, F. Biomimetics for next generation materials. *Philos. Trans. R. Soc. A Math. Phys. Eng. Sci.* **365**, 2907–2919 (2007). 10.1098/rsta.2007.0006 17855221

[14] Wegst, U. G. K. & Ashby, M. F. The mechanical efficiency of natural materials. *Philos. Mag.* **84**, 2167–2181 (2004).

[15] Jäger, I. & Fratzl, P. Mineralized collagen fibrils: A mechanical model with a staggered arrangement of mineral particles. *Biophys. J.* **79**, 1737–1746 (2000). 11023882 10.1016/S0006-3495(00)76426-5 PMC1301068

[16] Bhushan,B. Biomimetics: Bioinspired hierarchical-structured surfaces for green science and technology. *Springer Ser. Mater. Sci.*, **279**10.1007/978-3-319-71676-3 (2018).

[17] Manapat, J. Z., Chen, Q., Ye, P. & Advincula, R. C. 3D printing of polymer nanocomposites via stereolithography. *Macromol. Mater. Eng.* **302**, 1–13 (2017).

[18] Manapat, J. Z., Mangadlao, J. D., Tiu, B. D. B., Tritchler, G. C. & Advincula, R. C. High-strength stereolithographic 3D printed nanocomposites: Graphene oxide metastability. *ACS Appl. Mater. Interfaces* **9**, 10085–10093 (2017). 28230346 10.1021/acsami.6b16174

[19] Compton, B. G. & Lewis, J. A. 3D-printing of lightweight cellular composites. *Adv. Mater.* **26**, 5930–5935 (2014). 24942232 10.1002/adma.201401804

[20] Yu, W., Zhou, H., Li, B. Q. & Ding, S. 3D printing of carbon nanotubes-based microsupercapacitors. *ACS Appl. Mater. Interfaces* **9**, 4597–4604 (2017). 28094916 10.1021/acsami.6b13904

[21] Parwani, R. et al. Morphological and mechanical biomimetic bone structures. *ACS Biomater. Sci. Eng.* **3**, 2761–2767 (2017). 33418700 10.1021/acsbiomaterials.6b00652

[22] Dimas, L. S., Bratzel, G. H., Eylon, I. & Buehler, M. J. Tough composites inspired by mineralized natural materials: Computation, 3D printing, and testing. *Adv. Funct. Mater.* **23**, 4629–4638 (2013).

[23] Mirzaali, M. J. Multi-material 3D printing of functionally graded hierarchical soft–hard composites. *Adv. Eng. Mater.* **22**, 1901142. 10.1002/adem.201901142 (2020).

[24] Naghavi Zadeh, M. et al. Dynamic characterization of 3D printed mechanical metamaterials with tunable elastic properties. *Appl. Phys. Lett.* **118**(21), 211901. 10.1063/5.0047617 (2021).

[25] Wu, X., Meng, X. & Zhang, H. An experimental investigation of the dynamic fracture behavior of 3D printed nacre-like composites. *J. Mech. Behav. Biomed. Mater.* **112**, 104068 (2020). 32905921 10.1016/j.jmbbm.2020.104068

[26] Jia, Z. & Wang, L. 3D printing of biomimetic composites with improved fracture toughness. *Acta Mater.* **173**, 61–73 (2019).

[27] Chen, Q. & Pugno, N. M. Bio-mimetic mechanisms of natural hierarchical materials: A review. *J. Mech. Behav. Biomed. Mater.* **19**, 3–33 (2013). 23332379 10.1016/j.jmbbm.2012.10.012

[28] Gu, G. X., Takaffoli, M. & Buehler, M. J. Hierarchically enhanced impact resistance of bioinspired composites. *Adv. Mater.* **29**, 1–7 (2017). 10.1002/adma.201700060 28556257

[29] Curto, M., Kao, A. P., Keeble, W., Tozzi, G. & Barber, A. H. X-ray computed tomography evaluations of additive manufactured multimaterial composites. *J. Microsc.* **285**, 131–143 (2022). 34057229 10.1111/jmi.13034

[30] Kelly, A. & Tyson, W. R. Tensile fibre-reinforce11 and metals copper/tuxstes. *J. Mech. Phys. Solids* **13**, 329–350 (1965).

[31] S. T. Standard test method for apparent shear strength of single-lap-joint adhesively bonded metal specimens by tension loading (Metal-to-Method). **01**, 1–5 (2005).

[32] Barthelat, F. Nacre from mollusk shells: A model for high-performance structural materials. *Bioinspiration Biomimetics* **5**, 1–8 (2010). 10.1088/1748-3182/5/3/035001 20729573

[33] Croop, B. Astm d638 type iv. 44

[34] ASTM D3039 / D3039M – 08, *Standard test method for tensile properties of polymer matrix composite materials*, (ASTM International, West Conshohocken, PA, 2008). 10.1520/D3039_D3039M-08

[35] 14026, I. S. O. International Standard International Standard. *61010–1 © Iec2001* **2014**, 13 (2014).

[36] Swetly, T. et al. Bioinspired engineering polymers by voxel-based 3D-printing. *BioNanoMaterials* **17**, 145–157 (2016).

[37] 61851, I. International Standard International Standard. *61010–1 © Iec2001* **2006**, 13 (2006).

[38] Tran, P., Ngo, T. D., Ghazlan, A. & Hui, D. Bimaterial 3D printing and numerical analysis of bio-inspired composite structures under in-plane and transverse loadings. *Compos. Part B Eng.* **108**, 210–223 (2017).

